# Books

**Published:** 1882-03

**Authors:** 


### Books.

ILLUSTRATIONS OF DISSECTIONS IN A SERIES OF ORIGINAL COLORED PLATES, THE SIZE OF LIFE, REPRESENTING THE DISSECTION OF THE HUMAN BODY. By George Viner Ellis and G. H. Ford. (Reduced on a uniform scale, and reproduced in *fac simile*, expressly for Wood's Library of Standard Medical Authors.) Volume I. Second edition. New York: William Wood & Co. 1882. Wood's Library of Standard Medical Authors.

The title of this book clearly describes its character. It contains twenty-eight full page colored plates, accurately representing the original drawings.

The illustrations in this volume comprise views of the upper limb and of the head and neck.

The authors say: "With the view of carrying out the pictorial representation of dissections, the part of the human body to be illustrated is divided into suitable stages or regions; and the muscles, blood vessels and nerves of each region are shown in layers in the natural order of succession, so that their mutual connections may be brought before the eye at one and the same time." The object of printing the figures in colors is to make them as true representatives as possible of nature. The success which has rewarded the efforts of the publishers to produce these colors in the complicated figures, it seems to us, is all that could be desired. Concise and perspicuous descriptive texts accompany each plate.

---

MEMORANDA OF PHYSIOLOGY. By Henry Ashby, M.D., (Lond). New York. Wm. Wood & Co. 1882.

This little book (pocket size) of 319 pages, is offered only as a synopsis of the larger works on physiology. To those who are fond of such compends, this one will prove acceptable. The main facts of the science are accurately, though briefly, stated. The information is "founded on Quain's anatomy, Gray's anatomy, and Foster's text book of physiology."

---

THE STUDENT'S MANUAL OF VENEREAL DISEASES. By Berkely Hill and by Arthur Cooper. New York. Wm. Wood & Co. 1881. (Price 10 cents).

This may be termed a pamphlet, and contains 62 pages. The authors are physicians of eminence. Much valuable information is condensed into this short space. We are disposed, however, to criticise or review certain points.

We learn "the causes of syphilis are predisposing and exciting." Climate is offered as one of the predisposing causes. We must believe, though, that sexual passion is the omnipotent predisposing cause.

"In many persons syphilis ends spontaneously; the incurable cases are comparatively few; in the great majority the disease subsides completely at the end of the second year." We regard this as too rosy and hopeful a view for us to endorse.

"Cases in which mercury is appropriate—when the patient is in fair health etc." We believe mercury is nearly always appropriate in syphilis, unless in some very old cases; and not seldom in these.

Three pages are devoted to "chancre." This is intended for "chancroid," as it is termed in in this country. The confusion of terms is unfortunate; but the last named word, in place of "chancroid," is almost universal on the continent of Europe, and seems to have crept across the channel. Iodoform is extolled to the skies for these local venereal sores. Astringents are also recommended. Very correctly, caustics are almost ignored, except in phagedæna. Too much emphasis is laid upon pressure in the treatment of chancroidal bubo.

In urethritis, but little attention is given the most important of all measures, injections. In epididymitis, "painting the surface of the scrotum with collodion once daily, in cases of moderate severity, gives great relief." Our experience, we must say, fails altogether to endorse this plan of treatment; though the theory is nice enough.

We devote so much space to this little work, not because of its pretensions, but from the importance of the subject, and the probable large circulation of the book. There is much valuable instruction in it; but it is better appreciated by one already posted on the subject than by any other; and such a one may prefer an treatise more elaborate.

---

Suppression of Urine. Clinical Descriptions and Analysis of Symptoms. By E. P. Fowler, M.D. New York. Wm. Wood & Co. 1881.

This volume of 86 pages on the subject of anuria is peculiar in two respects: First, that one case, elaborately detailed, should serve as the text of the volume; second, the patience with which cases of suppression are collated from various sources and closely analyzed. Sixty-nine pages are occupied with this analysis.

The book is one to attract the attention of those interested in the subject. The reader may be especially surprised at the length of time (ten days) the patient lived, in the author's case, without any urine finding its way into the bladder. However, among the other cases reported, a yet longer duration of suppression, in several instances, is reported.

In the resume, a number of practical points are given; but these would have been more acceptable if the treatment had received some attention.

---

TRANSACTIONS OF THE MEDICAL ASSOCIATION OF GEORGIA. Thirty-second Annual Session, 1881. A. Sibley Campbell, M.D., Secretary.

This volume was mentioned in our last issue. It is with unalloyed pleasure that we mention its handsome binding, in cloth, and general appearance. It is, in this respect at least, far ahead of any previous issue, and superior, we believe, to the issue of any medical society of the United States.

In a prefatory note, the Secretary gives good reason for delay in publication. He urges the necessity for increased annual assessment that such delay may be prevented.

A number of wood-cuts and photo-engravings illustrate different articles. Portraits of Dr. C. W. Long and Robert Irvine are given.

In the proceedings, the most important action was upon the bill, as published, to be urged before the legislature for the regulation of the practice of medicine. This, though materially modified, has become a law.

The address of the president, Dr. J. C. Le Hardy, a sensible and practical paper, opens the appendix. The society took steps to carry out his suggestions as to prize essays.

Dr. R. J. Nunn contributes an article on female diseases, the result of errors in habit and hygiene during childhood and puberty. Dr. T. R. Wright, a paper, three cases of compound comminuted fracture of leg—recovery without suppuration. Dr. Kenan, physician at the lunatic asylum, has a case of Cut-throat. Dr. J. G. Hopkins gives some new uses of the fluid extract of ergot. Some interesting cases of wound penetrating the abdominal cavity are from the pen of Dr. W. H. Philpot. Dr. A. S. Campbell presents gunshot wound of abdomen, fecal fistula, spontaneous closure.

Dr. C. W. Hickman has a few pages on eye surgery. Dr. S. B. Hawkins details a rather remarkable case of miscarriage (at three and a half months) followed in five and a half months by labor at full term. Dr. De Saussure Ford, offers report of surgical cases, with discussion of quinine and tar water as antiseptics. Dr. S. H. Stout discusses dysmenorrhœa and puerperal convulsions. Dr. W. F. Westmoreland has an illustrated article on nævus.

The committee on necrology present memorial reports of Drs. W. J. Harrell, J. B. Hendrick, R. J. Bruce, J. C. Habersham, Easton Yonge, Robt. Irvine, T. F. Green, R. M. Smith, R. L. Roddy, C. W. Long, D. S. Brandon, W. R. Burgess, T. D. L. Ryan and W. C. Musgrove.

The memoir of Dr. Long is accompanied by an excellent photograph, and proofs of his claim to be the first surgeon to use ether for anesthesia.

We repeat, the make-up of the volume does credit to the care and energy of the Secretary. The papers, too, are above the average of State transactions. The Association and the profession of the State may be proud of the volume. It should give renewed interest in the prosperity of the Society. Extra copies may be obtained of the Secretary, at Augusta, at $1.50 per copy (postage fifteen cents extra).

---

Home and Climate Treatment of Pulmonary Consumption on the Basis of Modern Doctrines. By J. Hilgard Tyndale, M.D. New York: Bermingham & Co. 1882. Price 50 cents.

The contents of this little volume comprise five chapters, which treat respectively of the pathology of consumption; treatment in general; home treatment; climate treatment, and general conclusions.

The writer believes that defective nutrition—inability to repair lesions or waste, or to sustain the normal standard—is the starting point of consumption. He attempts to classify the pathology of the combined chronic, retrograde processes, which collectively make up the disease, by dividing them into classes and describing them as anatomical, clinical and historico-etiological.

His "home treatment" consists in the observance of well-known hygienic principles, with a view of improving the general nutrition, and in combatting untoward symptoms as they arise.

He very properly recommends the selection of a climate, when change is deemed advisable, which appears, from individual experiences, to be best suited to any given case. While general suggestions concerning the choice of a climate are made, no definite and invariable rule can be applied to all cases.

In the chapter on climate treatment, which occupies a large portion of the book, we are told that "Georgia is a state much less known than others climatically, and yet it is within its borders that I firmly believe a close approach to the ideal climate for consumptives can be found in more than one especial locality. * * * The benefit derived from the odor of pine trees is not to be denied. * * * Atlanta appears to me to be a suitable place, or, still more so, the village of Marietta, near the Kennesaw mountains, not far from Atlanta by rail, and 1,132 feet above sea level." Augusta is said to be the capital of Georgia.

The book is very carelessly written, and, among other defects, we note some inexcusable typographical errors.

A second edition of the monograph, thoroughly and carefully revised, will doubtless be a decided improvement upon the first.

